# TRADITIONAL AND NONTRADITIONAL INTERNSHIPS: A GERONTOLOGY STUDENT’S PERSPECTIVE

**DOI:** 10.1093/geroni/igad104.0490

**Published:** 2023-12-21

**Authors:** Jessica VanderWerf

**Affiliations:** University of South Florida, Tampa, Florida, United States

## Abstract

Today’s gerontology students are more diverse than ever. Some come to the classroom with little to no experience working with older adults, while others are looking for further education and/or credentials to add to their employment and career opportunities. Both traditional (e.g., nursing homes, senior centers) and non-traditional internship settings (e.g., research, teaching) are examined in regard to providing students with experiences that will enhance their knowledge, career development, and/or future academic goals. As both an undergraduate minor and a Master’s level gerontology student, the presenter discusses their own non-traditional internship experiences that helped them to reach their goal of pursuing a doctoral degree in aging studies. Feedback gathered from other students in a variety of internship types is also presented. Finally, lessons learned, finding the right fit, and the overall value of internships and similar experiences is discussed from a student perspective.

